# Phosphoinositide Recognition Sites Are Blocked by Metabolite Attachment

**DOI:** 10.3389/fcell.2021.690461

**Published:** 2021-07-22

**Authors:** Troy A. Kervin, Brittany C. Wiseman, Michael Overduin

**Affiliations:** ^1^Department of Biochemistry, University of Alberta, Edmonton, AB, Canada; ^2^Molecular and Cellular Biology, MacEwan University, Edmonton, AB, Canada; ^3^SMALP Network, Edmonton, AB, Canada

**Keywords:** lipid specificity, membrane recognition, phosphoinositide binding, lysine acetylation, arginine methylation, PX domain, protein regulation, metabolite signaling

## Abstract

Membrane readers take part in trafficking and signaling processes by localizing proteins to organelle surfaces and transducing molecular information. They accomplish this by engaging phosphoinositides (PIs), a class of lipid molecules which are found in different proportions in various cellular membranes. The prototypes are the PX domains, which exhibit a range of specificities for PIs. Our meta-analysis indicates that recognition of membranes by PX domains is specifically controlled by modification of lysine and arginine residues including acetylation, hydroxyisobutyrylation, glycation, malonylation, methylation and succinylation of sidechains that normally bind headgroups of phospholipids including organelle-specific PI signals. Such metabolite-modulated residues in lipid binding elements are named MET-stops here to highlight their roles as erasers of membrane reader functions. These modifications are concentrated in the membrane binding sites of half of all 49 PX domains in the human proteome and correlate with phosphoregulatory sites, as mapped using the Membrane Optimal Docking Area (MODA) algorithm. As these motifs are mutated and modified in various cancers and the responsible enzymes serve as potential drug targets, the discovery of MET-stops as a widespread inhibitory mechanism may aid in the development of diagnostics and therapeutics aimed at the readers, writers and erasers of the PI code.

## Introduction

Membrane readers are protein domains that recognize unique PI lipids that mark various organelle membranes found in eukaryotic cells. These conserved modules serve to reversibly recruit cytosolic proteins to membrane surfaces, thus controlling downstream assembly, signaling and trafficking events. The best understood are the FYVE, PH, and PX domain superfamilies, which represent the core of the PI code that underlies eukaryotic membrane recognition (Overduin et al., 2001; Sato et al., 2001). They comprise hundreds of domains and may only represent a small fraction of the total number of membrane readers (Overduin and Kervin, submitted), with the weak, dynamic or temperamental lipid binding activities of many proteins remaining technically difficult to detect. How they are regulated remains obscure, prompting this investigation into PI code control.

**FIGURE 1:**
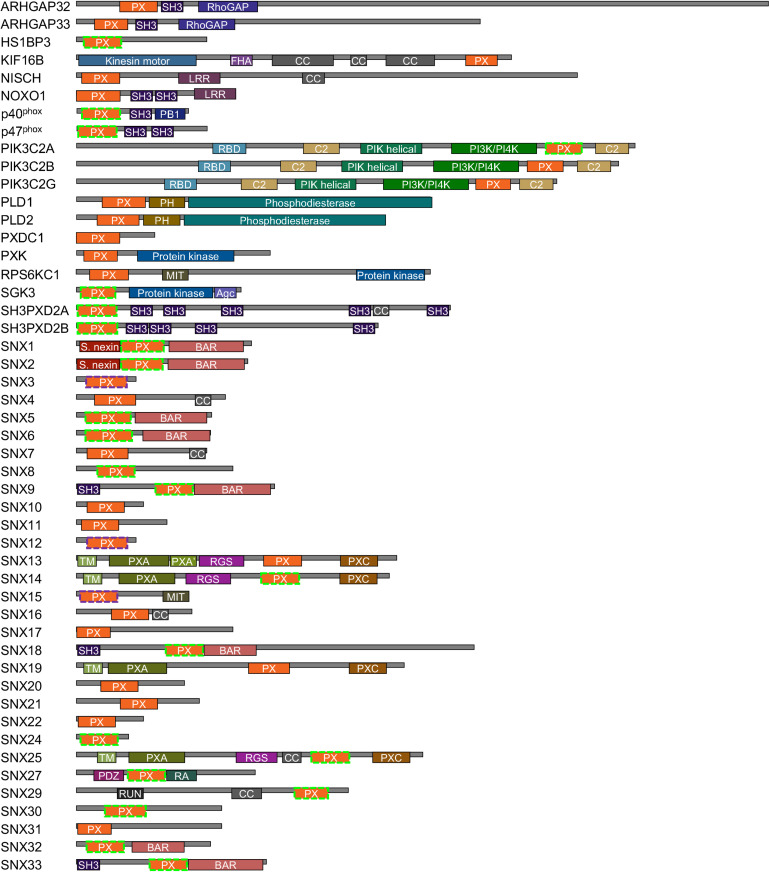
Human proteins containing PX domains. The PX domains that contain arginine and lysine-based MET-stops are bounded by dashed purple and green lines, respectively. Modules are color-coded and abbreviated as Agc (AGC-kinase C-terminal), BAR (Bin–Amphiphysin–Rvs), C2 (protein kinase C conserved region 2), CC (coiled coil), GAP (GTPase-activating protein), FHA (forkhead-associated), LRR (leucine-rich repeat), MIT (microtubule interacting and transport), PB1 (Phox and Bem1), PDZ (Postsynaptic density 95, Disk large, Zonula occludens), PH (pleckstrin homology), PIK (phosphoinositide 3-kinase), PXA (PX-associated), PXC (PX C-terminal), RA (Ras-associating), RBD (Ras binding domain), RGS (regulator of G protein signaling), RUN (RPIP8, unc-14 and NESCA), SH3 (src homology 3) and TM (transmembrane) domains.

Of the diverse families of membrane readers, PX domains are uniquely able to detect the full spectrum of seven phosphoinositide phosphate (PIP) signals (Table 1). This superfamily includes up to ∼120 members per genome across fungi, protists, viridiplantae, and metazoa (Banerjee et al., 2010), with 49 distinct members in *Homo sapiens* (Figure 1). The family of human PX domains is the focus here as it comprises the best-defined group of membrane readers in terms of 3D structures, ligand specificities and membrane interactions. Moreover, since the discovery of residues that, when phosphorylated, phosphorylation, block PIP recognition (PIP-stops) in three sorting nexins (Lenoir et al., 2018), this family is ideal for investigating how other protein modifications could control membrane recognition.

**TABLE 1:** Human PX domain properties.

Protein	LSI	PIP ligands	MSS	PSS	MAI	Expression	PDB	References
ARHGAP32	8	3,4,5	0	5	W	9.4	IT	Hayashi et al., 2007; Chandra et al., 2019
ARHGAP33	0	0	0	0	N	22.7	IT	Chiang et al., 2003; Chandra et al., 2019
HS1BP3	5	3,34,35,45,345	2	0	S	8.4	IT	Holland et al., 2016; Chandra et al., 2019
KIF16B	6	3,34,45,345	0	1	S	6.2	2v14	Blatner et al., 2007; Pyrpassopoulos et al., 2017; Chandra et al., 2019
NISCH	8	3,34	0	6	W	83.0	3p0c	Lim and Hong, 2004; Chandra et al., 2019
NOXO1β	9	45,345	0	0	W	0.2	2l73	Cheng and Lambeth, 2004; Ueyama et al., 2007; Davis et al., 2012
NOXO1γ	7	4,5,35	0	2	W	0.1	IT	Cheng and Lambeth, 2004; Ueyama et al., 2007
p40phox	10	3	2	1	S	9.3	1h6h	Bravo et al., 2001; Ellson et al., 2001; Kanai et al., 2001; Chandra et al., 2019
p47phox	6	3,34,45,345	1	6	S	25.9	1kq6	Ago et al., 2001; Kanai et al., 2001; Chandra et al., 2019
PIK3C2α	8	34,35,45	2	1	S	19.0	2ar5	Song et al., 2001; Stahelin et al., 2006; Chandra et al., 2019
PIK3C2β	8	34,45,345	0	2	S	9.7	IT	Song et al., 2001; Chandra et al., 2019
PIK3C2γ	7	34,35,45,345	0	0	W	1.0	2wwe	Chandra et al., 2019
PLD1	10	345	0	1	S	5.4	IT	Du et al., 2003; Stahelin et al., 2004; Lee et al., 2005
PLD2	10	45	0	16	W	26.9	IT	Sciorra et al., 1999; Lee et al., 2005; Mahankali et al., 2013; Han et al., 2020
PXDC1	nd	nd	0	0	nd	69.6	IT	
PXK	10	3	0	4	W	18.9	IT	Takeuchi et al., 2010; Chandra et al., 2019
RPS6KC1	6	3,34,45,345	0	6	S	9.9	IT	Hayashi et al., 2002; Chandra et al., 2019
SGK3	8	3,34	7	2	S	31.3	1xte	Virbasius et al., 2001; Xu J. et al., 2001; Chandra et al., 2019
SH3PXD2A	10	3	1	1	S	34.5	IT	Abram et al., 2003; Chandra et al., 2019
SH3PXD2B	8	3,34	3	6	S	19.3	IT	Abram et al., 2003; Buschman et al., 2009
SNX1	10	34	6	15	S	35.5	2i4k	Cozier et al., 2002; Zhong et al., 2002; Carlton et al., 2004; Catimel et al., 2008; Ceccato et al., 2016; Chandra et al., 2019
SNX2	10	34	13	17	S	43.9	IT	Zhong et al., 2002; Carlton et al., 2005; Catimel et al., 2008; Chandra et al., 2019
SNX3	10	3	8	13	S	169.4	5f0j	Xu Y. et al., 2001; Zhong et al., 2002; Ceccato et al., 2016; Lenoir et al., 2018; Chandra et al., 2019
SNX4	10	3	0	3	W	22.0	IT	Traer et al., 2007; Chandra et al., 2019
SNX5	6	3,34,35,45	4	1	W	34.4	3hpc	Merino-Trigo et al., 2004; Liu et al., 2006; Catimel et al., 2009; Koharudin et al., 2009; Chandra et al., 2019
SNX6	10	4	9	2	W	29.4	IT	Niu et al., 2013; Chandra et al., 2019
SNX7	10	3	0	3	W	18.0	IT	Xu Y. et al., 2001; Chandra et al., 2019
SNX8	10	3	3	0	S	11.0	IT	Dyve et al., 2009; van Weering et al., 2012
SNX9	6	3,34,45,345	5	4	W	42.5	2raj	Lundmark and Carlsson, 2003; Pylypenko et al., 2007; Yarar et al., 2007; Yarar et al., 2008; Chandra et al., 2019
SNX10	10	3	0	0	W	10.6	4on3	Chandra et al., 2019
SNX11	1	3,4,5,34,35,45,345	0	0	S	16.5	4ikb	Xu et al., 2013; Chandra et al., 2019
SNX12	10	3	2	8	S	41.3	2csk	Pons et al., 2012; Ceccato et al., 2016; Chandra et al., 2019
SNX13	8	3,34	0	3	W	7.9	IT	Zheng et al., 2001; Mas et al., 2014; Chandra et al., 2019
SNX14	0	0	4	1	N	19.5	IT	Mas et al., 2014; Chandra et al., 2019
SNX15	3	3,4,34,35,45,345	3	0	S	26.9	IT	Danson et al., 2013; Chandra et al., 2019
SNX16	10	3	0	2	S	5.8	5gw0	Hanson and Hong, 2003; Ceccato et al., 2016; Xu J. et al., 2017; Chandra et al., 2019
SNX17	10	3	0	9	S	89.3	IT	Knauth et al., 2005; Czubayko et al., 2006; Chandra et al., 2019
SNX18	9	34,45	2	0	S	12.8	IT	Haberg et al., 2008; Nakazawa et al., 2011; Liebl et al., 2017
SNX19	10	3	0	2	S	26.4	IT	Mas et al., 2014; Chandra et al., 2019
SNX20	5	3,5,35,45	0	2	S	3.2	IT	Schaff et al., 2008; Clairfeuille et al., 2015
SNX21	8	3,45	0	6	S	32.1	IT	Clairfeuille et al., 2015
SNX22	6	3,34,45,345	0	2	S	9.4	2ett	Song et al., 2007; Chandra et al., 2019
SNX24	6	3,34,35,45	1	7	S	8.1	4az9	Chandra et al., 2019
SNX25	7	34,35,45,345	5	2	S	12.9	5woe	Mas et al., 2014; Chandra et al., 2019
SNX27	10	3	1	6	S	19.9	4has	Lunn et al., 2007; Ghai et al., 2011; Rincon et al., 2011; Chandra et al., 2019
SNX29	7	3,34,45	2	0	S	8.0	IT	Chandra et al., 2019
SNX30	10	3	3	1	W	6.3	IT	
SNX31	10	3	0	1	S	0.5	IT	Vieira et al., 2014; Chandra et al., 2019
SNX32	0	0	2	3	N	4.9	6e8r	Chandra et al., 2019
SNX33	9	34,45	1	9	S	20.8	IT	Almendinger et al., 2011; Ma and Chircop, 2012

**FIGURE 2:**
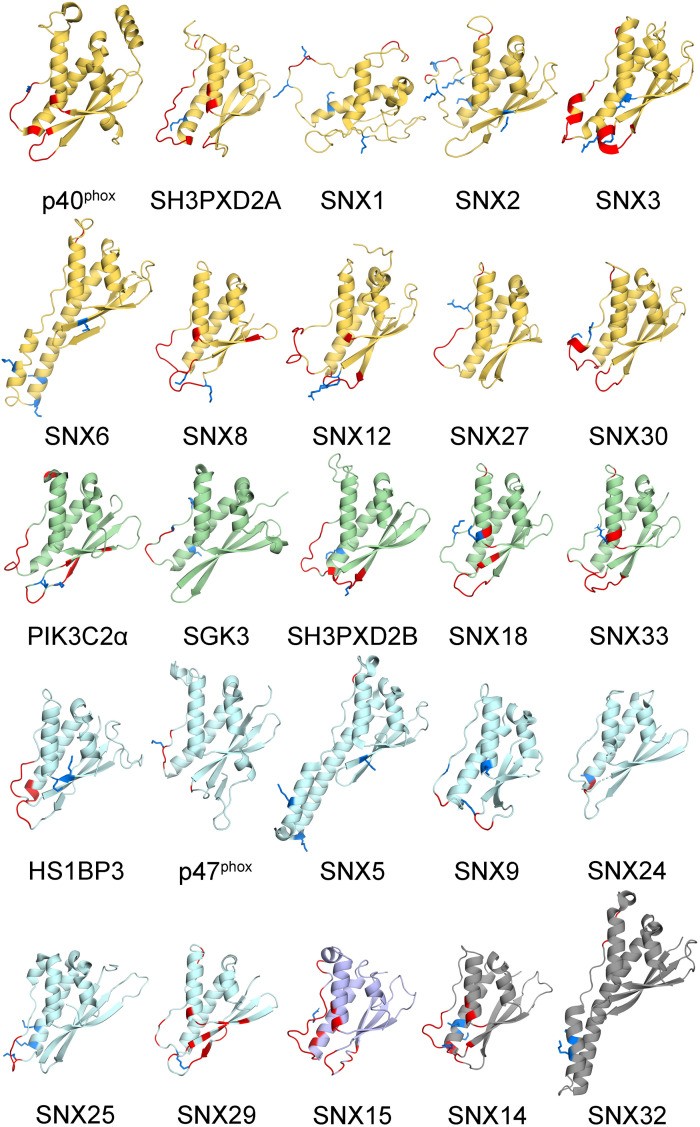
Human PX domain structures with MET-stops. Metabolite-modified Arg and Lys residues are shown with sidechains in blue. Membrane docking residues with MODA scores greater than 30 are shown in red. Backbone ribbon colors correspond to the specificity indices of 10 (yellow), 8–9 (green), 5–7 (light blue), 1–4 (purple), and non-binding (gray).

*The metabolite-stop score (MSS), PIP-stop score (PSS), Membrane Affinity Index (MAI), Lipid Specificity Index (LSI), and PIP ligands are indicated for each domain. Also listed are the PDB structures starting with the one used in Figure 2, with I-TASSER derived structures denoted “IT.” PIP ligands are denoted by phosphate positions, e.g., PtdIns (3,5)P_3_ is denoted “35,” while “nd” and “0” indicate not determined or non-binding, respectively. Average expression levels are in units of transcripts per million (GTEx Consortium, 2020).*

The regulation of membrane readers could conceivably occur by several different mechanisms. A variety of PIP kinases and phosphatases add and remove phosphates from the lipid’s inositol ring, and are located in different parts of the cell (Balla, 2013). However, altering PIP levels would influence many effector proteins rather than controlling individual players. Coincidence detection allows membrane readers to be recruited only when multiple partners can be simultaneously engaged (Carlton and Cullen, 2005). However, this does not address how the original protein which anchors a larger complex gathers on membranes in the first place. Proteins including those on membranes are downregulated by ubiquitination, but this can be a slow process (Vietri et al., 2020). Protein kinases and phosphatases regulate the lipid-binding activities of membrane readers by activating and deactivating PIP-stops (Lenoir et al., 2018; Kervin and Overduin, 2021). However, the possibility that other modifications control PIP-binding remains unexplored. The addition of metabolites to Arg and Lys residues is of particular interest as these residues are conserved and critical determinants of PIP recognition (Cheever et al., 2001).

Arginine and lysine sidechains are also frequent sites of covalent attachment of metabolites. These post-translational modifications (PTMs) include the addition of acetyl, butyryl, glucosyl, glutaryl, malonyl, methyl, and succinyl groups, which can have profound effects on diverse signaling processes (Figlia et al., 2020). These events are known to regulate protein–protein interactions and cellular metabolism as well as being correlated with progression of cancer and diabetes. However, their role in regulating protein-membrane interactions remains surprisingly unexplored.

Here we report a proteome-wide meta-study of the control of PX domains by metabolite-based modifications of Lys and Arg residues, revealing that such MET-stops are surprisingly frequent in membrane binding sites and are structurally geared to interfere with PIP interactions. Patterns in the distribution of MET-stops are similar to those observed with PIP-stops and are found across eukaryotes, suggesting a conserved developmental function. This supports a widespread role for metabolite modification in toggling PIP binding by membrane readers to regulate local biological events throughout the cell.

## Materials and Methods

### Comparing PX Domain Sequences

The sequences of human PX domain-containing proteins and their homologs were obtained from UniProt (UniProt, 2019). The sequences were aligned using Clustal Omega (Sievers and Higgins, 2018) and adjusted manually to line up structurally and functionally critical residues, and visualized using Jalview 2 (Waterhouse et al., 2009). Point mutations were identified from the cBioPortal (Cerami et al., 2012) and COSMIC (Tate et al., 2019) databases and references therein.

### Membrane Site Definition

The 3D structures of PX domains were calculated with the I-TASSER program to perform structural analysis of the entire human superfamily (Yang et al., 2015). The model with the highest cluster size was chosen as the representative structure. The available PX domain structures in the RCSB Protein Data Bank (PDB) (Berman et al., 2000) were compared and the highest resolution entry with defined membrane binding residues was selected where there were several entries. The structural models were visualized using PyMOL and ICM (Neves et al., 2012) software, and residues that are membrane-interactive and modified were compared. Each PX domain’s membrane binding surface was predicted with the Membrane Optimal Docking Area (MODA) program (Kufareva et al., 2014). This generated a score for each residue in each structure to indicate its likelihood of interacting with membranes, with scores exceeding 30 predicting a probable role in membrane docking. The data from all human PX domains was compared to map the consensus membrane docking sites, which are located in the loop connecting the first and second β strands, in the third β strand and at the beginning of the first α helix, and in the loop encompassing the proline-rich (PR) element and second α helix. This consensus binding area was compared to the experimentally determined PIP-binding modes from NMR and crystal structures of PX domains bound to bicelles, micelles and phospholipids (Table 1). The individual MODA scores of all residues in all three consensus binding sites were added to generate each domain’s total MODA score. For NMR structures, a representative model of the ensemble was selected with MODA scores for such residues nearest to the mean. All PTMs were shown on the alignment and those occurring within the three sites were considered candidate MET-stops as they are positioned to compromise cognate membrane interactions.

### Mapping Modifications

A standardized measure of confidence that membrane binding by a domain is modulated by metabolite-based modifications was needed. We propose a MET-stop score (MSS), which quantifies this likelihood based on existing data, and complements the PIP-stop score (PSS), which relates to Ser/Thr/Tyr phosphorylation occurrences in membrane binding sites (Lenoir et al., 2018; Kervin and Overduin, 2021). Relevant protein modifications based on experimental data were obtained from cBioportal (Cerami et al., 2012), dbPTM (Lee et al., 2006), iProteinDB (Hu et al., 2019), iPTMnet (Huang et al., 2018), PhosPhAT (Heazlewood et al., 2008), PhosphoGrid (Stark et al., 2010), PhosphoSite (Hornbeck et al., 2015), PLMD (Xu H. et al., 2017), PTMcode2 (Minguez et al., 2015), qPTM (Yu et al., 2019), SuperPhos databases (Lanz et al., 2021), and related studies (Hansen et al., 2019), providing broad proteome coverage. Each instance of a candidate MET-stop in the consensus membrane binding sites contributed to a domain’s total MSS value. These modified residues were also compared to the membrane binding residues identified in PIP-complexed PX domains of Grd19, p40^phox^, p47^phox^, SNX3, SNX9, and SNX11 PX domains (Bravo et al., 2001; Karathanassis et al., 2002; Zhou et al., 2003; Pylypenko et al., 2007; Stampoulis et al., 2012; Lenoir et al., 2018; Xu et al., 2020) to investigate whether they could compromise ligand recognition. To optimize the MSS we tested various sequence and spatial distance bounds around the MODA maxima and spans in which modified Lys or Arg residues can be considered candidate MET-stops. We opted for sequence alignment-based boundaries that encompass the binding elements consistently identified by MODA as this was determined to be the most accessible approach. All candidate MET-stops added to the MSS of a domain. Each unique study reporting a metabolite-based modification of a Lys or Arg residue in any of the three sites added 1 to the MSS, while modifications with 2–4 references added 2, and modifications with 5 or more references added 3. This scoring function is intended to reduce bias due to varying tissue-specific protein expression levels by integrating and standardizing PTM data from many studies. Manual curation of the collated data ensured that each original dataset is only cited once per modification. Hence the MSS indicates the relative frequency of metabolite-based modifications of a domain that could modulate membrane binding and allows for the comparison of Lys and Arg modifications with PIP-stops as well as PIP specificities and membrane affinities.

### Lipid Specificity and Affinity

The ligand specificities of PX domains were quantified using a broadly applicable Lipid Specificity Index (LSI). A domain with relatively high affinity for only a single PIP was assigned a LSI value of 10. In contrast, PX domains that displayed no discernible binding to any PIP were given LSI values of 0. All other PX domains were given an intermediate LSI value equal to 10 minus 1 for each additional in-class PIP ligand and minus 2 for each additional PIP ligand not in their class. Each of the seven PIPs were divided into two classes depending on whether they had 1 or over 1 terminal phosphates, respectively, and PX domains were assigned to the same class as their predominate ligands. Hence a LSI of 1 indicates a perfectly non-specific domain that binds all seven PIPs. The data from all published reports of lipid binding by each PX domain were analyzed to generate a database of PX domain lipid specificities and membrane affinities. In cases where there was conflicting evidence, we gave precedence to quantitative data utilizing liposomes and lipid-binding assays. The subcellular localization of PX domains to organelle membranes was also considered as support for binding to relevant PIPs. The membrane affinity index (MAI) was used to classify the membranes affinities of PX domains as strong, weak and none (S, W, or N) based on evaluation of the relevant studies showing approximately nanomolar affinity, micromolar affinity or no binding to lipid bilayers containing cognate PIP ligands, respectively.

### Statistical Analysis

Statistical tests were applied following the collection of MSS, PSS, LSI and expression levels for all human PX domains, as described above. Linear regression, correlation and paired *t*-tests were performed using JMP 15 (JMP Version, 2019) in order to investigate potential relationships between scores (*P* < 0.05). Candidate MET-stops and PIP-stops were assigned values from 1 to 3, LSI was scaled from 1 to 10 and average expression levels were obtained (GTEx Consortium, 2020). A linear regression was performed to calculate whether MSS or PSS could be predicted based on LSI, or whether MSS or PSS could be predicted based on expression level. An additional regression test was performed to see whether MSS could predict PSS in order to enhance our understanding of how metabolite-based modifications might be involved in membrane binding. Pairwise correlation tests were applied to further test for positive or negative associations between each set of variables, thus providing information about which variables might be influencing the ability of membrane protein readers to bind PIP ligands. Finally, a paired *t*-test was performed to determine whether the proportion of modified arginine and lysine residues is higher inside the membrane recognition sites of PX domains as opposed to outside these sites (*P* < 0.05).

## Results

### Structural Basis of Membrane Binding

The membrane binding and post-translational modification sites in the structures of all human PX domains were compared. This involved using I-TASSER to calculate 29 structures, all of which had acceptable qualities with average TM and confidence scores of 0.81 ± 0.09 and 0.38, respectively (Table 1). The remaining PX domains had complete 3D structures from previous NMR spectroscopy and X-ray crystallography studies. As expected, the PX structures converged well and display a common fold and membrane binding area (Cheever and Overduin, 2004; Figure 2). This common structural framework provides a straightforward basis for comparing the various lipid binding and regulatory properties.

All human PX domains were categorized based on their PIP ligands using a lipid specificity index (LSI) in order to investigate possible relationships with metabolite-based PTMs (Table 1). The twenty domains which specifically recognize only a single type of membrane-bound PIP were assigned the maximum LSI value of 10. Three domains exhibit no detectable membrane binding activity and were given a LSI of 0. Those domains with intermediate lipid specificities were assigned LSI values equal to 10–n_1_–2n_2_, where n_1_ is the number of PIP ligands of the same class and n_2_ is the number of PIPs ligands of a different class (see section “Materials and Methods”). The LSI values of the PX domains of SNX30 and SNX33, which have not been experimentally determined, were inferred to be similar to that of SNX7 and SNX18, respectively, as they have the most similar sequences, binding motifs and multidomain structures. Altogether, this represents the best-defined and most diverse family of membrane readers in terms of PIP-binding, allowing us to explore which types of PIP interactions are likely to be affected by the various protein modifications.

In order to investigate how PIP recognition might be regulated, we structurally defined the relevant binding sites using the MODA algorithm, which pinpoints all residues in protein structures that are likely to interact with membranes (Kufareva et al., 2014). These sites were consistent with experimentally mapped contacts from complexed structures of PX domains of Grd19, p40^phox^, p47^phox^, SNX3 and SNX9 proteins (Bravo et al., 2001; Karathanassis et al., 2002; Zhou et al., 2003; Pylypenko et al., 2007; Stampoulis et al., 2012; Lenoir et al., 2018). Three elements that are commonly involved in binding PIP-containing membranes were identified. Site 1 is a membrane-insertion loop that connects the β1 and β2 strands, with the exception of SNX5, SNX6, and SNX32 where it instead forms a protein binding element (Paul et al., 2017). Site 2 contains the canonical regulatory PIP-stop that was discovered in SNX1, SNX3, and SNX12 (Lenoir et al., 2018). In the case of the PX domain of p47^phox^ this site is particularly extensive, being capable of binding additional acidic phospholipids in addition to a PIP molecule (Karathanassis et al., 2002; Stampoulis et al., 2012; Takeuchi et al., 2012). Site 3 spans a long irregular loop that can form a secondary PIP-binding site (Chandra et al., 2019) and includes a proline rich element (PRE), which can also serve as a SH3 domain docking site (Hiroaki et al., 2001; Ago et al., 2003). These three sites constitute a common PIP-binding surface that is found across the superfamily and includes dynamic loops and an accessible set of basic and hydrophobic sidechains. These features are known to attract proteins to membranes where they stereospecifically recognize PIP headgroups and insert stably into phospholipid bilayers, thus endowing PX domains with the ability to read PI codes.

### Analysis of Membrane Binding Properties

Membrane reader function is associated with a domain’s affinity and specificity for lipid ligands including PIP molecules and is subject to regulatory influences. The ligand affinities of PX domains varies widely, which is to be expected given their diverse biological roles and multidomain architectures (Figure 1). Each PX domain was assigned to the membrane affinity index (MAI) based on the available literature. There are 31, 15 and 3 PX domains with strong, weak and no apparent affinity for membranes, respectively. PIP specificity diverges across the family, with 20, 11, 13, and 2 PX domains that exhibit absolute, high, medium and low membrane selectivity, respectively, based on LSI values of 10, 9–8, 7–5, and 4–1. The lipid specificities and membrane affinities of human PX domains are not significantly correlated based on the currently available experimental data. This indicates that these properties are essentially independent dimensions of function which are tailored to various protein, pathway and organelle needs. We next explored whether these functional properties could be subject to control by various types of PTMs.

### Modifications of Membrane Binding Sites

The discovery that phosphorylating a conserved serine in the PIP binding sites of SNX1, SNX3, and SNX12 PX domains blocks membrane recruitment led to the concept of a PIP-stop (Lenoir et al., 2018; Kervin and Overduin, 2021). These are residues that, when phosphorylated, compromise binding to PIP-containing membranes. While this concept has since been independently validated with SNX1 (Feng et al., 2020), it has not been extended to other types of modifications, prompting a deeper analysis here. Mapping of all the known acetylation, butyrylation, glycation, malonylation, methylation and succinylation sites to the PX structures revealed that there are 87 residues in membrane-binding sites with such modifications, of which 51 are found in human proteins (Table 2).

**TABLE 2:** PX domains contain diverse metabolite-based post-translational modifications.

Protein	Residue	PTM	Species	Site	Protein	Residue	PTM	Species	Site
HS1BP3	K69	Butyrylation	*H. sapiens*	FLVSkKYSE	SNX3	R45	Methylation	*H. sapiens*	VGRGrFTTY
HS1BP3	K70	Butyrylation	*H. sapiens*	LVSKkYSEI	SNX3	R45	Methylation	*M. musculus*	VGRGrFTTY
p40^phox^	K92	Acetylation	*X. tropicalis*	ELPPkIFVG	SNX3	R45	Methylation	*R. norvegicus*	VGRGrFTTY
p40^phox^	K92	Acetylation	*M. musculus*	TLPAkVYMG	SNX3	R45	Methylation	*B. taurus*	VGRGrFTTY
p40^phox^	K92	Acetylation	*D. rerio*	TLPGkVFMG	SNX3	R45	Methylation	*C. elegans*	VGKMrYTDY
p40^phox^	K92	Acetylation	*B. taurus*	TLPAkVYVG	SNX3	R45	Methylation	*D. rerio*	VGRNrFTTY
p47^phox^	K79	Acetylation	*H. sapiens*	LPAPkWFDG	SNX3	R45	Methylation	*X. tropicalis*	VGRGrYTTY
PIK3C2A	K1434	Acetylation	*H. sapiens*	FTYHkKYNP	SNX3	R70	Methylation	*M. musculus*	TVRRrYSDF
PIK3C2A	K1440	Acetylation	*H. sapiens*	YNPDkHYIY	SNX5	K46	Acetylation	*H. sapiens*	RDKVkFTVH
SGK3	K71	Acetylation	*H. sapiens*	AMALkIPAK	SNX5	K108	Acetylation	*H. sapiens*	EKMQkLGEG
SGK3	K71	Acetylation	*M. musculus*	AMALkIPAK	SNX5	K118	Acetylation	*H. sapiens*	GSMTkEEFA
SGK3	K71	Succinylation	*M. musculus*	AMALkIPAK	SNX6	K47	Acetylation	*H. sapiens*	RDKVkFTVH
SGK3	K75	Acetylation	*H. sapiens*	KIPAkRIFG	SNX6	K109	Acetylation	*H. sapiens*	EKLQkLGEG
SGK3	K75	Methylation	*H. sapiens*	KIPAkRIFG	SNX6	K119	Acetylation	*H. sapiens*	GSMTkEEFT
SGK3	K88	Acetylation	*H. sapiens*	PDFIkQRRA	SNX6	K124	Acetylation	*H. sapiens*	EEFTkMKQE
SH3PXD2A	K92	Acetylation	*H. sapiens*	DVAVkRLKP	SNX8	K85	Acetylation	*H. sapiens*	LIPEkKGLF
SH3PXD2B	K17	Acetylation	*H. sapiens*	LDVQkRRVP	SNX8	K91	Acetylation	*H. sapiens*	GLFLkHVEY
SH3PXD2B	K93	Acetylation	*H. sapiens*	DVAVkRLIP	SNX9	K267	Methylation	*H. sapiens*	MYGLkSYIE
SNX1	K184	Acetylation	*B. taurus*	HFAVkRRFS	SNX9	K288	Acetylation	*H. sapiens*	NHRYkHFDW
SNX1	K221	Acetylation	*B. taurus*	IGMTkVKVG	SNX9	K288	Malonylation	*H. sapiens*	NHRYkHFDW
SNX1	K226	Acetylation	*B. taurus*	VKVGkEDSS	SNX9	K313	Malonylation	*H. sapiens*	SLPDkQVTG
SNX1	K237	Acetylation	*H. sapiens*	EFLEkRRAA	SNX12	R44	Methylation	*B. taurus*	VGVGrARFT
SNX1	K237	Acetylation	*R. norvegicus*	EFLEkRRAA	SNX12	R46	Methylation	*B. taurus*	VGRArFTTY
SNX1	K237	Acetylation	*M. musculus*	EFLEkRRAA	SNX14	K648	Acetylation	*H. sapiens*	IIGPkNYEF
SNX1	K167	Acetylation	*C. elegans*	SALTkTKTN	SNX14	K654	Acetylation	*H. sapiens*	YEFLkSKRE
SNX1	K358	Acetylation	*D. rerio*	MGMTkVKVG	SNX14	K656	Acetylation	*H. sapiens*	FLKSkREEF
SNX1	K363	Acetylation	*D. rerio*	VKVGkEDPS	SNX15	R81	Methylation	*M. musculus*	PAFPrAQVF
SNX2	K181	Acetylation	*H. sapiens*	EFSVkRRFS	SNX18	K314	Malonylation	*H. sapiens*	HRRYkHFDW
SNX2	K181	Acetylation	*M. musculus*	EFSVkRRFS	SNX18	K338	Malonylation	*H. sapiens*	HLPEkQATG
SNX2	K181	Butyrylation	*H. sapiens*	EFSVkRRFS	SNX24	K69	Acetylation	*H. sapiens*	NWVPkVLEQ
SNX2	K211	Malonylation	*H. sapiens*	PAPEkSIVG	SNX25	K584	Acetylation	*H. sapiens*	KLPFkSIDQ
SNX2	K211	Acetylation	*H. sapiens*	PAPEkSIVG	SNX25	K589	Acetylation	*H. sapiens*	SIDQkFMEK
SNX2	K218	Acetylation	*H. sapiens*	VGMTkVKVG	SNX25	K593	Acetylation	*H. sapiens*	KFMEkSKNQ
SNX2	K223	Acetylation	*H. sapiens*	VKVGkEDSS	SNX27	R218	Methylation	*H. sapiens*	FTFPrLPGK
SNX2	K234	Acetylation	*H. sapiens*	EFVEkRRAA	SNX29	K729	Acetylation	*M. musculus*	AIGNkDAKF
SNX2	K234	Succinylation	*H. sapiens*	EFVEkRRAA	SNX30	K153	Acetylation	*D. rerio*	KFVMkGVVD
SNX3	R39	Methylation	*S. pombe*	HGIGrNMFT	SNX30	K158	Acetylation	*H. sapiens*	PLPEkFVVK
SNX3	R43	Methylation	*H. sapiens*	VGVGrGRFT	SNX30	K162	Acetylation	*H. sapiens*	KFVVkGVVD
SNX3	R43	Methylation	*M. musculus*	VGVGrGrFT	SNX30	K171	Acetylation	*X. tropicalis*	KFVVkGVVD
SNX3	R43	Methylation	*R. norvegicus*	VGVGrGRFT	SNX32	K100	Glycation	*H. sapiens*	ASREkLQKL
SNX3	R43	Methylation	*B. taurus*	VGVGrGRFT	SNX32	K103	Glycation	*H. sapiens*	EKLQkLGEG
SNX3	R43	Methylation	*D. rerio*	VGVGrNRFT	SNX33	K243	Acetylation	*X. tropicalis*	YRRYkHFDW
SNX3	R43	Methylation	*X. tropicalis*	IGVGrGRYT	SNX33	K245	Acetylation	*D. rerio*	YRRYkHFDW

*The residues and sequence motifs with MET-stops in the three membrane binding sites of PX domains from proteins of various species are listed, with the central, modified residue of the motifs in lower case. Butyrylation includes hydroxyisobutyrylation.*

**FIGURE 3:**
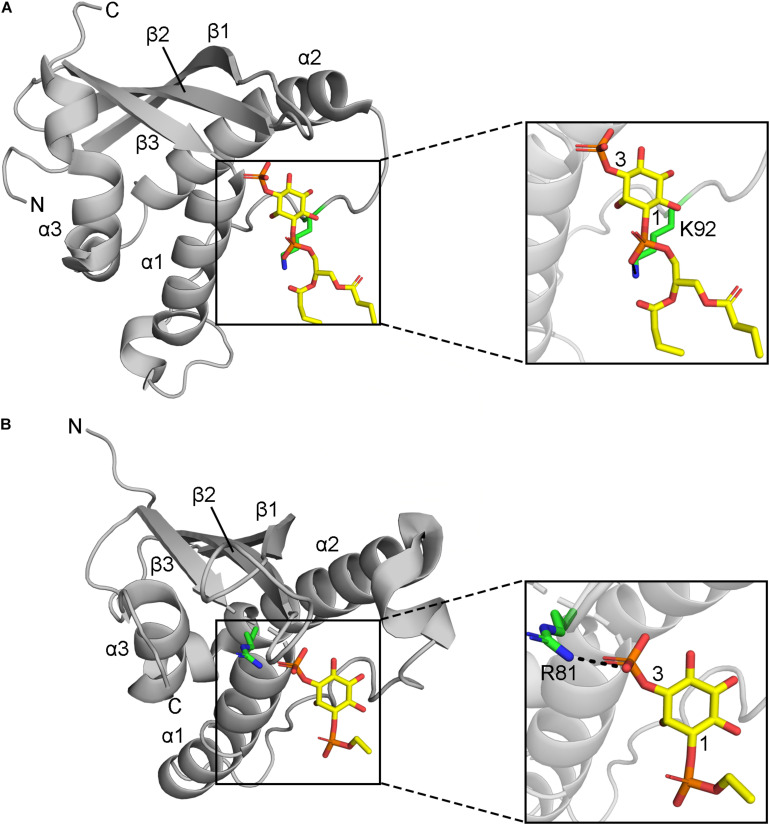
Positions of MET-stop residues in PIP-complexed PX domain structures. **(A)** The p40^phox^ K92 sidechain in Site 3 hydrogen bonds with the 1-phosphate and is acetylated. **(B)** Grd19 R81 sidechain in Site 2 hydrogen bonds with the 3-phosphate in PI3P and the equivalent residue in SNX3 is methylated.

Residues that directly contact lipid headgroups and insert into membrane mimics are heavily modified, including in the membrane insertion loop (MIL), PIP-binding RRY motif in the β3 strand, KxLF motif after the PRE, and RR element in the α2 helix (Figure 3). Modifications in these places would directly interfere with hydrogen bonding and electrostatic interactions of Arg and Lys residues that normally mediate phospholipid recognition and membrane attraction while introducing steric impediments to lipid headgroup docking.

**FIGURE 4:**
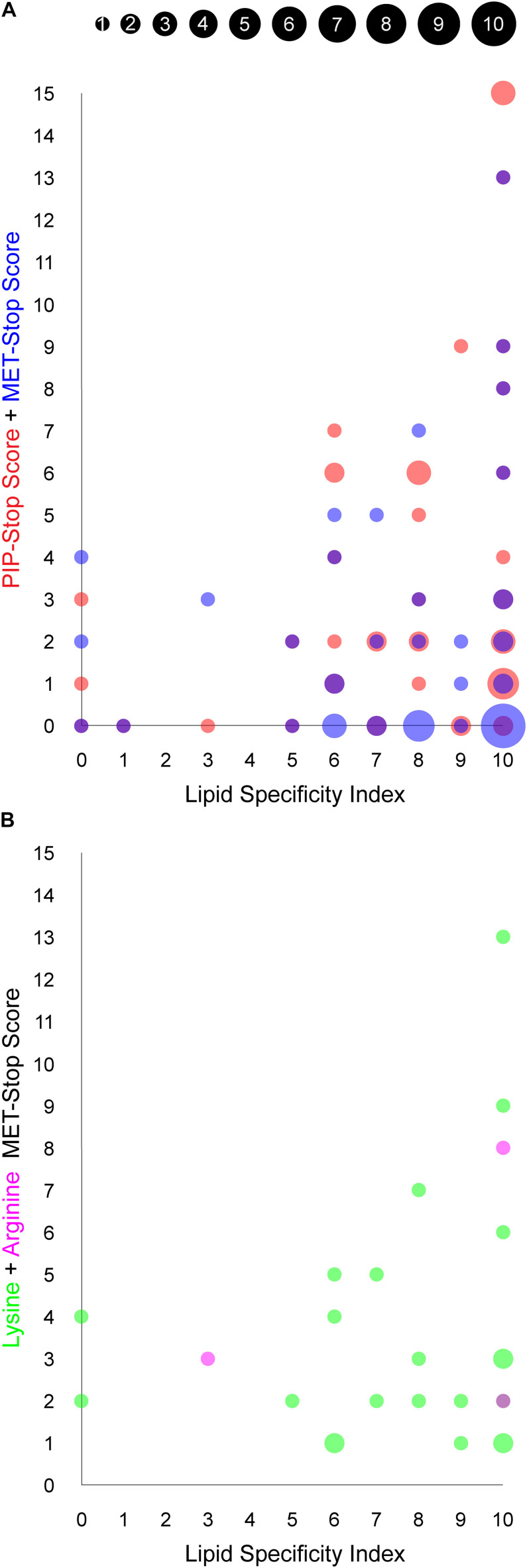
Relationship between PIP binding specificity and post-translational modifications of PX domains. The area of each circle is proportional to the number of PX domains that occupy the position of the circle, as indicated in the inset and are based on values in Table 1. **(A)** The PIP-stop score (PSS) and MET-stop score (MSS) of each human PX domain is plotted against its Lipid Specificity Index (LSI), shown in red and blue, respectively, while overlapping circles appear purple. **(B)** The contributions to the MSS from lysine and arginine modifications for each PX domain, discounting scores of zero, are shown in green and magenta, respectively, while overlapping circles appear purple.

A paired *t*-test was performed to determine if there is a significant difference in the relative proportion of modified arginine and lysine residues within membrane binding sites compared to those outside such sites (Figure 4B). The data revealed that the mean proportion of modified residues is significantly higher inside sites than outside sites (*t* = –3.2360, df = 49, *P* = 0.0022). Arginine modifications are rarely reported, yet are found in sorting nexins 3, 12, and 15 (which do not exhibit any lysine modifications) and are exclusively located in guanidinium groups which are positioned to contact phospholipid headgroups directly. In contrast lysine acetylations are found in other PX domains where they are more widely distributed, while protein phosphorylations offer more frequent modifications that are typically adjacent to points of stereospecific PIP contacts. Thus, we propose that each residue-specific modification offers a differentiated handle for complementary erasure of membrane reader functions.

The membrane binding sites of PX domains are modified in many species, suggesting conserved regulatory mechanisms. For example, the RGR motif in the β1-β2 loop of SNX3 is heavily methylated in human, mouse and rat cells and also modified in frog, bull, nematode, yeast and zebrafish homologs. The conserved PIP-coordinating Lys residue after the PRE is modified in p40^phox^, SGK3, SNX2, SNX9, SNX18, and SNX30, while Lys residues N-terminal to the PIP-binding RR motif in α2 are modified in SGK3, SH3PXD2A, SH3PXD2B, SNX1, SNX2, SNX6, SNX14, and SNX25. Hence membrane binding elements and proximal residues appear to be preferentially modified throughout evolution. This suggests a longstanding mechanism to regulate to PX domains by abolishing or weakening canonical PIP-membrane recognition through metabolite-based inhibition.

### Influences on Modification Frequency

We explored whether Arg and Lys modifications in PX domains are related to PIP-stops and ligand specificities using the MSS, PSS and LSI parameters, respectively. Higher MSS values appear visually to be concentrated in PX domains with high LSI values, reminiscent of the pattern observed with PSS and LSI (Kervin and Overduin, 2021, Figure 4A). A significant linear regression equation was found between MSS and PSS [*F*(1, 48) = 8.3586, *P* = 0.0058, *R*^2^ = 0.148311], where the predicted PSS is equal to 2.5472 + 0.5939 (MSS). A correlation test also indicated that MSS and PSS are related [*r*(48) = 0.3851, *P* = 0.0058]. Together this suggests that cells may generally be inclined to regulate specific PX domains with both MET-stop and PIP-stop mechanisms.

Although PIP-stops are more likely to be found in PX domains with high specificity, a pronounced relationship between MET-stops and lipid specificity has not yet emerged. A significant linear regression equation was found between PSS and LSI [*F*(1, 47) = 5.1535, *P* = 0.0278, *R*^2^ = 0.0988], where the predicted PSS is equal to 0.0904 + 0.4735 (LSI), indicating that lipid specificity is a predictor of PIP-stops. In contrast, a linear regression equation relating MSS and LSI indicates that MET-stops are not related to lipid specificity (*P* > 0.05). Likewise, correlation tests indicated that PSS and LSI are related [*r*(48) = 0.3143, *P* = 0.0278] while MSS and LSI are not (*r* = 0.0809, *P* = 0.5803). A caveat is that MET-stops are 2.5-fold less abundant than PIP-stops. Hence their analysis would benefit by inclusion of richer data on such membrane reader modifications. Arg-based MET-stops are particularly rare yet are highly abundant in SNX3 and are conserved in SNX12. This suggests that arginine methylation may have evolved to control whether SNX3 and its close relative SNX12 localize to endosomal membranes, while lysine modifications could serve to regulate PX domains more broadly inside the cell with less regard for lipid specificity. Thus both Arg- and Lys-based of MET-stops appear to differentially complement PIP-stops, which serve to more frequently and selectively down-regulate the most PIP-specific domains.

Several factors could influence susceptibility of membrane readers to metabolite modification. The highest MSS values were found in SGK3 and sorting nexins 1, 2, 3, and 6, which selectively recognize only PI3P, PI4P or PI(3,4)P_2_ lipids, suggesting that endosomal and Golgi compartments are likely locations for metabolite attachment. The PX proteins that are uniquely plasma membrane localized, that is, NOXO1β, PLD1 and PLD2, lack any reported MET-stops in their PX domains, suggesting that this environment may be less fertile for such modifications. Two PX proteins, SNX14 and SNX32, do not bind membranes yet contain candidate MET-stops and PIP-stops, suggesting that some of these PTMs may influence events other than PIP-mediated membrane interactions. A further 19 PX domains exhibit intermediate MET-stop scores and display a wide range of PIP specificities, although all visit endocytic routes. In contrast, 20 membrane-binding PX domains do not exhibit known metabolite-based modifications, with 17 of these containing PIP-stops instead. The remainder including sorting nexins 10 and 11 may not be regulated by such modifications *in vivo* or could mediate membrane interactions that are constitutive or dependent on coincidence detection.

The broad dynamic range of the expression of PX domain-containing proteins is also a factor. We found that their average mRNA expression levels, as shown in Table 1, could be predicted by both MSS [*F*(1, 48) = 7.3181, *P* = 0.0094, *R*^2^ = 0.1323] and PSS [*F*(1, 48) = 14.7109, *P* = 0.0004, *R*^2^ = 0.2346]. The predicted expression is equal to either 16.9500 + 3.9156 (MSS) or 12.3204 + 3.2080 (PSS). Correlation tests further revealed that expression is related to both MSS [*r*(48) = 0.3637, *P* = 0.0094] and PSS [*r*(48) = 0.4843, *P* = 0.0004]. This suggests that cells tend to regulate their most highly expressed PX domains through such PTMs, although this relationship is not absolute. For example, the PX domains of highly expressed NISCH and SNX17 do not exhibit any discernable MET-stops despite contributing to endocytic pathways, although they do carry PIP-stops. In contrast, the highly expressed PXDC1 lacks a known function or any such PTMs yet is an established tumor marker in endothelial cells (St Croix et al., 2000). Thus, the presence of a PTM that perturbs a lipid binding site is influenced by not only the activity of the responsible enzymes but also membrane reader expression level and co-localization in the context of a network of regulatory pathways that are only beginning to be mapped and understood.

## Discussion

The discovery of MET-stops exposes a new dimension of ways by which cells could regulate their diverse membrane interactions. These events are carried out by enzymes including methyl and acyl transferases, while a complementary set of demethylases and deacetylases such as HDACs and sirtuins can remove such modifications. While historically these PTMs have been thought to regulate histone interactions with DNA (Figlia et al., 2020), here we show that they are also frequently positioned to regulate recognition of PI codes by a large number of membrane readers. Undoubtably many more pathways are governed by such mechanisms, although some modifications will represent metabolic noise with no functional consequences, necessitating careful structure-based assessment. The preferential modification of lipid binding sites revealed here suggests broad selective pressure to control PIP recognition, with membrane reader sites representing significant biological targets. This is consistent with earlier studies showing that such modifications control the subcellular localization and activity of proteins (Sundaresan et al., 2011; Thandapani et al., 2013; Su et al., 2017). In addition to metabolite addition, ubiquitination of lysine residues also occurs in PX domains and is found predominantly in and C-terminal to membrane binding Site 3. While generally thought to maintain quality control of proteins including sorting nexins (Hanley and Cooper, 2020), such ubiquitin-based modifications would also alter lipid interactions *via* proximal membrane docking surfaces. The presence of a terminal acetyl group prevents ubiquination from occurring on a lysine and promotes protein stability (Caron et al., 2005). Thus MET-stops could promote the formation of relatively long-lived reservoirs of negatively regulated PI readers which are dislodged from membrane surfaces, although functional studies would be needed to validate this hypothesis.

**FIGURE 5:**
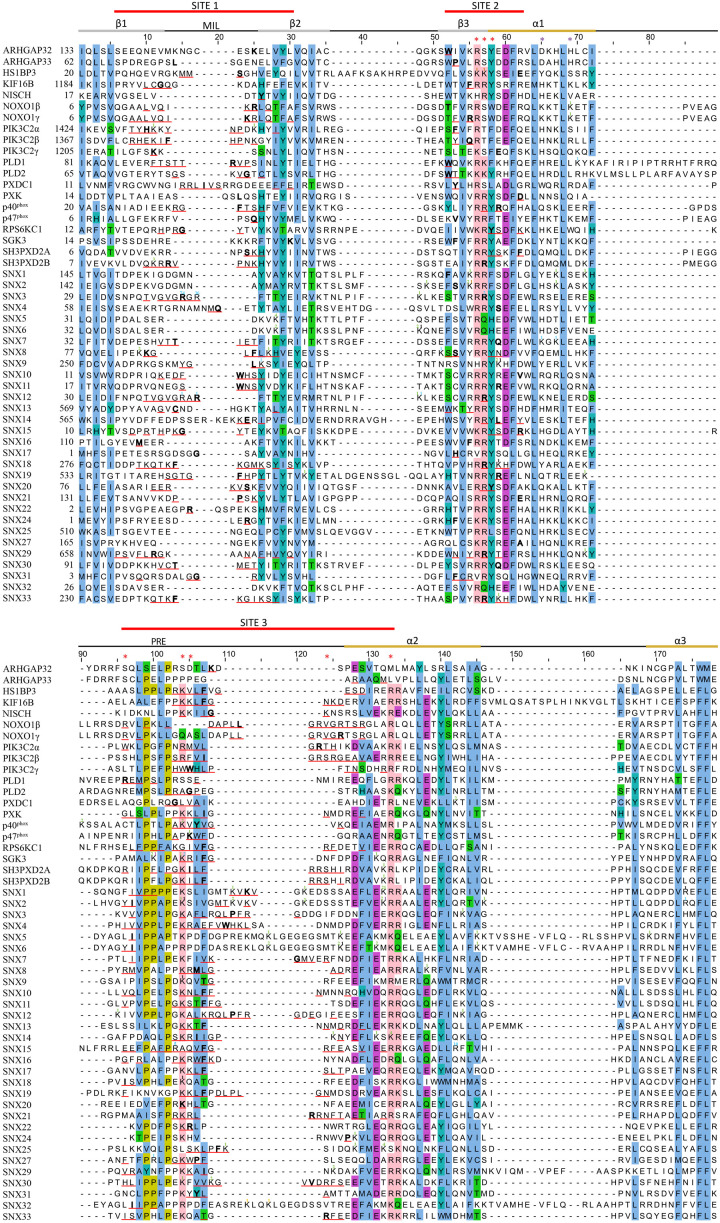
Sequence alignment of all human PX domains. Citations of acetylated, butyrylated, glycated, malonylated, methylated and succinylated residues are indicated with a green, brown, orange, black, blue and purple superscript, respectively. The secondary structure, membrane insertion loop (MIL) and proline rich element (PRE) are indicated below the three red-lined membrane docking sequences. PIP headgroup and phosphatidic acid binding positions are indicated with red and purple asterisks, respectively. The residue with the highest MODA score in each site is bolded. Aligned conserved hydrophobic, proline, polar, acidic, and basic residues are highlighted in blue, olive, green, purple, and pink.

The putative regulatory mechanism mediated by MET-stops appears to be highly conserved across many membrane readers. For example, the presence of a conserved and methylated glycine-arginine-rich (GAR) motif (Thandapani et al., 2013) in SNX3 homologs (Table 2) could infer the presence of an ancient and critical switch. The most recurrent MET-stop in human PX domains occurs at a Lys residue immediately C-terminal to the PRE (Figure 5), indicating that this is a dominant regulatory feature of the superfamily. Interestingly this feature has been supplanted by an Arg-based MET-stop in SNX15, suggesting regulatory convergence. The diversity of modification types across the three Sites of this superfamily suggests that several enzymatic pathways evolved in parallel to directly toggle residues responsible for lipid recognition. While this could be taken to suggest redundant control of crucial PI reader functions, the complexity of the actual patterns indicate complementary roles of each distinct enzyme-mediated regulatory pathway.

There are likely candidates responsible for MET-stop activation. These include protein arginine methyltransferases such as PRMT8, which localizes to the plasma membrane through myristoylation (Toma-Fukai et al., 2016), as well as lysine acetylases that are known to block membrane interactions of the Akt kinase by modifying its PH domain (Sundaresan et al., 2011). Proteins that promote lysine acetylation and organelle biogenesis engage sorting nexins at endosomes in plants and mammals, suggesting longstanding interactions (Zhang et al., 2014). The enzymes controlling these PTMs are emerging as cancer targets, and with clinical trials of inhibitors underway, there is a need to clarify their mechanisms of action (Dang and Wei, 2021; Samuel et al., 2021). The data presented here indicate that negative regulation of endosome recruitment of SNX3 and its associated retromer assembly (Lucas et al., 2016; Leneva et al., 2021) would be blocked by methyl transferase inhibitors, while a broad range of membrane reader interactions could be affected by acetyltransferase and deacetylase inhibitors. Disease-linked mutations found at MET-stop residues now have more predictable effects. For example, various mutations in human PX domains have been associated with malignancies (Tate et al., 2019; Table 3). As these point mutations alter MET-stop motifs they are positioned to block or deregulate PIP binding activity, inferring potential signaling or trafficking defects that could contribute to pathogenic effects.

**TABLE 3:** Cancer-linked point mutations involving MET-stop motifs of human PX domains.

Malignancy	Associated mutations involving PX domain MET-stops
Brain	SNX12 R44C, SNX15 R81W, SNX25 S594P
Breast	SNX3 R45L
Colon	SGK3 R76I, SH3PXD2A K17T, SNX1 R185G, SNX2 K223N, SNX3 S72R, SNX5 F47C, SNX15 R81W, SNX18 R312H
Endometrioid	SNX6 V46I, SNX9 R286G, SNX12 R46C, SNX27 P217H
Esophageal	SNX29 K729T
Liver	p47^phox^ K79R, SNX6 Q108L, SNX8 K85E, D166G
Lung	SNX9 R286W
Stomach	SNX1 R238W, SNX2 R235W, SNX12 R46C, SNX24 R103L
Skin	SNX3 R43L, SNX24 R103Q, SNX30 P156L
Thyroid	SNX5 K44E

*The original studies are cited in COSMIC database (Tate et al., 2019).*

Other domains and proteins may be impacted by the discovery of MET-stops. Larger assemblies including those with pathogen proteins (Paul et al., 2017) and retromers (Lucas et al., 2016; Leneva et al., 2021) are anchored by PX domain binding to PIPs and could be dislodged. Multi-subunit complexes are also influenced by phosphorylation events that alter protein-protein interactions (Ago et al., 2003), and some of the metabolite-based PTMs identified here could also affect protein-protein interactions rather than solely influencing membrane binding. The presence of so many MET-stops in the PX superfamily suggests that thousands of other membrane readers (Overduin and Kervin, submitted) could also utilize such regulatory mechanisms, including the hundreds of FYVE and PH domains that also recognize PIPs (Lenoir et al., 2015; Eitzen et al., 2019). While their unique folds, binding sites and lipid specificities will require further analysis, it appears that direct control of PI code readers by a growing variety of specialized PTMs could provide a unifying principle for ensuring high-fidelity membrane recognition in any eukaryotic cell.

## Data Availability Statement

The datasets presented in this study can be found in online repositories. The names of the repository/repositories and accession number(s) can be found in the methods section of this article.

## Author Contributions

BCW, MO, and TAK collected, analyzed the data, and wrote the manuscript. All authors contributed to the article and approved the submitted version.

## Conflict of Interest

The authors declare that the research was conducted in the absence of any commercial or financial relationships that could be construed as a potential conflict of interest.
